# Assessment of the Impact of Trace Essential Metals on Cancer Development

**DOI:** 10.3390/ijms25136842

**Published:** 2024-06-21

**Authors:** Aleksandra Górska, Agnieszka Markiewicz-Gospodarek, Mateusz Trubalski, Marta Żerebiec, Julia Poleszak, Renata Markiewicz

**Affiliations:** 1 Department of Normal, Clinical and Imaging Anatomy, Medical University of Lublin, 4 Jaczewskiego St., 20-090 Lublin, Poland; aleksandragorska@umlub.pl; 2 Students Scientific Association, Department of Normal, Clinical and Imaging Anatomy, Medical University of Lublin, 4 Jaczewskiego St., 20-090 Lublin, Poland; mateusztrub@gmail.com (M.T.); zerebiecm22@gmail.com (M.Ż.); juliapoleszak3@gmail.com (J.P.); 3 Occupational Therapy Laboratory, Chair of Nursing Development, Medical University of Lublin, 4 Staszica St., 20-081 Lublin, Poland; renatamarkiewicz@umlub.pl

**Keywords:** heavy metals, zinc, copper, cobalt, iron, manganese, carcinogenesis

## Abstract

This study examines the impact of zinc, copper, cobalt, iron, and manganese on cancer development, considering their dual roles as potential promoters or inhibitors within tumorigenesis. A comprehensive analysis of existing literature and experimental data is conducted to elucidate the intricate relationship between these trace elements and cancer progression. The findings highlight the multifaceted effects of zinc, copper, cobalt, iron, and manganese on various aspects of cancer development, including cell proliferation, angiogenesis, and metastasis. Understanding the nuanced interactions between these trace elements and cancer could offer crucial insights into tumorigenesis mechanisms and facilitate the identification of novel biomarkers and therapeutic targets for cancer prevention and treatment strategies. This research underscores the importance of considering the roles of essential trace elements in cancer biology and may ultimately contribute to advancements in precision medicine approaches for combating cancer.

## 1. Introduction

Carcinogenesis is a complex and protracted progression characterized by a series of stages. The conventional understanding posits that the development of cancer comprises a triphasic process, encompassing initiation, promotion, and progression stages. It encompasses genetic mutations, genomic instability, heightened activity of oncogenes, suppression of tumour suppressor genes, alterations in genetic material, and aberrations in cellular metabolism [1,2]. The initiation and progression of carcinogenesis are influenced by exogenous and endogenous factors, coupled with individual elements such as genetic predisposition [3,4]. It can be inferred that the amalgamation of diverse risk factors exerts the most significant impact on the evolution of cancer [4].

An extremely important group of exogenous carcinogenesis factors is the group of heavy metals (HMs). In biological systems, heavy metals have been documented to impact various cellular organelles and constituents, including the cell membrane, mitochondria, lysosomes, endoplasmic reticulum, nuclei, and certain enzymes associated with metabolic processes, detoxification mechanisms, and damage repair pathways. Metal ions have been identified to engage with cellular components like DNA and nuclear proteins, inducing DNA damage and structural alterations that can result in carcinogenesis [5,6].

Heavy metals such as arsenic, cadmium, chromium, and nickel are well-documented carcinogens, but the focus of this article will be on the tumorigenic effects of copper, cobalt, iron, zinc, and manganese. These metals are not only essential for various biological functions but also pose a risk of toxicity and carcinogenesis when present in abnormal concentrations. Copper is known for its role in angiogenesis and metastasis [7,8], while cobalt has been implicated in hypoxia signalling pathways that can promote tumour growth [9]. Iron, through its participation in the Fenton reaction, can generate reactive oxygen species (ROS) leading to oxidative stress and DNA damage [10,11]. Zinc plays a crucial role in DNA synthesis and repair but can also influence cancer progression depending on its concentration and cellular context [12]. Manganese, while essential for enzymatic functions, has been associated with neurotoxicity and potential carcinogenic effects through mechanisms that are still being elucidated [13].

The aim of this article is to compare the impact of selected heavy metals on carcinogenesis. We will focus on tumorigenesis induced by copper, cobalt, iron, zinc, and manganese. Our goal is to gather and summarize information on the role of these metals in cancer development, as they have not been collectively discussed in this context before. This review seeks to summarize how these essential, yet potentially harmful elements contribute to the complex process of carcinogenesis.

## 2. Methods and Search Criteria 

To conduct a literature review on carcinogenesis and the relationship with heavy metals, the following steps were undertaken. 

### 2.1. Defining the Scope and Objectives 

The review aimed to interstrand the mechanisms of carcinogenesis, with a particular focus on the role of heavy metals. Specific heavy metals of interest included: zinc, copper, cobalt, iron, and manganese. 

### 2.2. Search Strategy 

Comprehensive searches were conducted in multiple scientific databases, including PubMed, Scopus, and Web of Science. Keywords used for the search included combinations of terms such as: carcinogenesis, heavy metals, cancer, metal toxicity, zinc, copper, cobalt, iron and manganese.

### 2.3. Inclusion Criteria 

Peer-reviewed articles, studies published within the last 20 years, research focusing on the biological mechanisms of heavy metals in carcinogenesis, and reviews or meta-analyses on the topic. 

### 2.4. Exclusion Criteria

Articles not available in English, studies with insufficient data on heavy metals, and research focused on non-cancerous outcomes. 

Each study was assessed for methodological quality including sample size, study design, statistical analysis, and potential biases. High-quality studies were given more weight in the synthesis of findings. 

Data from the selected studies were synthesized to identify common terms and patterns regarding the role of heavy metals in carcinogenesis. Mechanisms of action, such as DNA damage, oxidative stress, and disruption of cellular signaling pathways, were highlighted. Differences in the carcinogenic potential of different heavy metals were also discussed. 

## 3. The Influence of Specific Heavy Metals on the Carcinogenic Process 

### 3.1. Zinc 

The indispensability of zinc (Zn) for human physiology was conclusively determined in 1963. Over the last five decades, remarkable progress has been witnessed in both the clinical and fundamental aspects of understanding zinc metabolism in humans [8]. The human body’s mass contains approximately 2–3 g of zinc, with skeletal muscle and bone accounting for around 57% and 29% of the total zinc content, respectively. The heart and blood plasma are recognized to contain 0.4% and 0.1% of the body’s zinc, respectively. Insufficient dietary intake, reduced absorption, or elevated zinc loss can lead to a state of deficiency [10]. Reduced serum zinc levels have been observed in various cancer patients, encompassing those with breast and prostate cancers [11].

Zinc, a micronutrient essential for all living organisms, plays a crucial role in various biochemical pathways within human cells. One of these roles is that Zn forms associations with more than 2500 proteins, representing approximately 10% of the total human proteome [10] and assumes a vital role as a structural constituent within structural motifs termed “zinc fingers”, present in diverse RNA and DNA binding proteins. In this capacity, zinc contributes to the preservation of structural integrity for a substantial portion of these proteins [10,12]. Zinc is also indispensable for diverse enzyme activities, gene expression, and critical cellular functions, including cellular proliferation. A noteworthy transcription factor activated by zinc is the metal response element-binding transcription factor-1 (MTF-1), a protein featuring six zinc fingers and multiple domains. Metal regulatory transcription factor-1 (MTF-1) functions as a zinc sensor, regulating the expression of genes pivotal for zinc homeostasis and providing protection against metal toxicity and oxidative stress (OS) [12]. Oxidative stress and heightened inflammatory cytokines are acknowledged as significant contributing factors in numerous age-associated chronic diseases, mutagenesis, and cancer [8].

**Figure 1:**
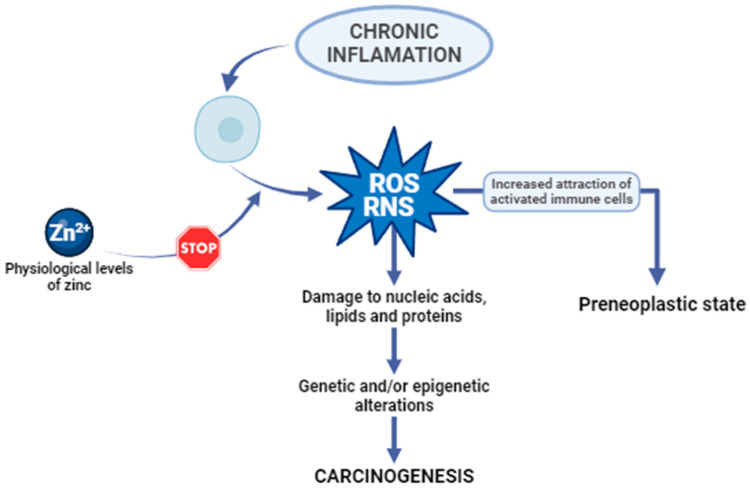
The anticarcinogenic effect of zinc. ROS, reactive oxygen species. RNS, reactive nitrogen species [14,15].

Oxidative stress is characterized by an imbalance between the generation of free radicals and reactive metabolites, commonly known as oxidants or reactive oxygen species (ROS), and their removal through protective mechanisms termed antioxidants. Reactive oxygen species are natural by-products of cellular metabolism, serving essential roles in activating signaling pathways within animal cells in response to alterations in intra- and extracellular environmental conditions [13]. In cells experiencing chronic inflammation, the substantial release of ROS and reactive nitrogen species (RNS) attracts an increased number of activated immune cells, thereby amplifying dysregulated processes, and culminating in a preneoplastic state. If the production of cellular ROS/RNS surpasses the endogenous antioxidant response, irreversible oxidative damage to nucleic acids, lipids, and proteins may occur, inducing genetic and/or epigenetic alterations that disrupt the regulation of oncogenes and tumour suppressor genes. The processes of oxidative stress and chronic inflammation are intricately linked, and the inability to inhibit these processes may result in genetic/epigenetic changes that initiate carcinogenesis (Figure 1) [14]. This illustrates how important it is to maintain the correct level of zinc in the body. Physiological levels of zinc demonstrate an inhibitory effect on the generation of reactive oxygen species, encompassing superoxide anion (·O^−^), hydrogen peroxide (H_2_O_2_), and hydroxyl radical (OH·), as well as reactive nitrogen species, including peroxynitrite. The direct antioxidant function of zinc ions is associated with their interaction with thiol groups, thereby shielding them from oxidative processes. Zn serves as a cofactor for the antioxidant enzyme Cu, Zn-superoxide dismutase (SOD1), and its activity is attenuated in conditions of zinc deficiency. Furthermore, studies indicate that zinc may indirectly modulate the functionality of other antioxidant enzymes [15]. 

As mentioned earlier, diminished serum zinc levels have been observed in various cancer patients, including those with breast, prostate, and endometrial cancers (Table 1) [16].

Due to the increased incidence of cancer in recent years, an attempt was made to conduct in vitro tests. In the case of gastric cancer, a high frequency of mutations has been associated with PI3K-Akt-mTOR signaling pathways, and genetic changes in this pathway have had a direct impact on the progression of many cancers [17]. In the above study, the created piperine-loaded ZnO nanocomposite (ZnO-Pip-NC) was found to have anticancer activity in the case of gastric cancer [17]. 

**Table 1:** Characteristics of breast, prostate, and endometrial cancers.

Type of Cancer	Characterization	Ref.
Breast cancer (BC)	Breast cancer, the most common cancer in women worldwide, has diverse types based on hormone and human epithelial growth factor receptor 2 (HER2) status: luminal A/B, HER2-positive, and triple-negative (TNBC). Zinc imbalance is linked to breast cancer, with low serum zinc but high zinc in cancer tissues. Zinc plays a crucial role in cancer progression, affecting cell transformation and tumor aggressiveness by influencing zinc transporters.	[16,18,19]
Prostate cancer (PCa)	Prostate cancer ranks as the second most common cancer in men globally, with high mortality rates, especially in cases with extracapsular disease. Unlike normal and benign prostate tissue, malignant prostate tissue shows decreased zinc levels, indicating a role for zinc alterations in cancer development. Zinc concentrations drop early in prostate cancer progression, inhibiting citrate oxidation, a key function of prostate cells. This loss of zinc may remove its inhibitory effects on cancer cells, potentially promoting prostate cancer initiation and progression.	[20,21]
Endometrial cancer (EC)	Endometrial cancer is a type of cancer that originates in the lining of the uterus known as the endometrium. Research has examined zinc metabolism in different cancers, including endometrial cancer. Although a direct link between serum zinc levels and endometrial cancer risk or progression hasn’t been established, studies suggest zinc may influence pathways related to cancer development and progression. Zinc is thought to possess anti-cancer properties by aiding in DNA repair, regulating cell growth, and impacting immune function. However, further research is required to comprehensively grasp the connection between zinc levels and endometrial cancer.	[22]

### 3.2. Copper 

#### 3.2.1. Copper’s Biological Role 

Copper (Cu) plays a crucial role as a vital micronutrient in various fundamental biological processes [23]. The primary source of copper for individuals is typically through their diet, with organ meats and shellfish being among the most copper-rich food options. The recommended daily intake of copper for adults is advised to be within the range of 0.8–2.4 mg/day [24]. Although only small amounts of copper are necessary in our diet, an ample supply of this metal is essential to support the growth and development of the human body [25]. It engages in various biological processes, such as lipid metabolism, energy regulation, and the synthesis of neurotransmitters [26]. In normal conditions, effective homeostatic mechanisms maintain a low concentration of intracellular copper ions [27]. However, an imbalance in cellular metal ion levels, whether in excess or deficiency, can be equally detrimental, as it may lead to an intensified rise in oxidative stress in both scenarios [28]. An abundance of copper ions induces heightened cellular respiration, leading to cytotoxic effects and eventual cell death as the levels progressively surpass a critical threshold [27]. Copper plays a substantial role in altering the function of specific types of superoxide dismutase (SOD) isoforms and other enzymes, including ceruloplasmin (CP). These enzymes are either directly or indirectly engaged in maintaining the balance of redox homeostasis [28]. Ensuring a balance of copper ions in biological systems is crucial to preventing atherosclerosis and cardiovascular diseases associated with it [29]. Disruption in the regulation of copper levels is also believed to play a role in the process of carcinogenesis [30]. The concentrations of copper ions can function as a vital indicator of cancer progression [27]. Irregular accumulation of copper is noticeable in various malignant tumours, and a connection has been identified between increased copper levels in both serum and tissues and the development of multiple types of cancers [31]. Studies on lung cancer, prostate cancer, breast cancer, stomach cancer, and thyroid cancers have identified a notable elevation in serum copper ion levels among tumour patients compared to those without tumours [32]. Additionally, a substantial increase in copper concentration was detected in the serum, bile, and gallbladder tissue of individuals diagnosed with gallbladder carcinoma [33]. 

#### 3.2.2. Copper in Cancer

**Figure 2:**
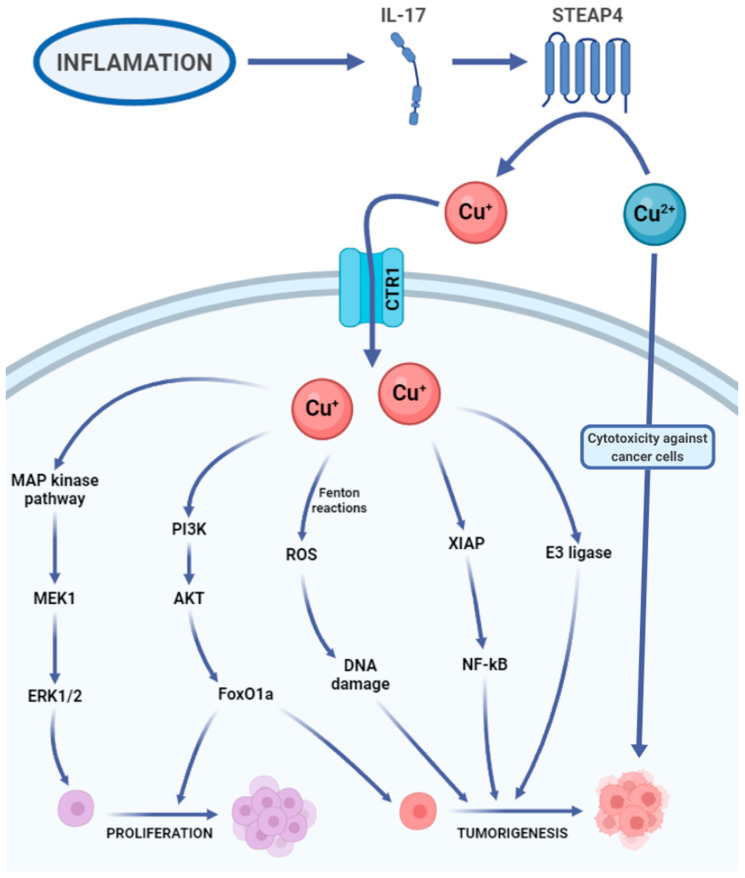
Copper’s carcinogenic potential. IL-17, interleukin 17. Cu^+^, Cu^2+^, copper ions. CTR1, high-affinity copper uptake protein 1 (copper transporter 1). MAP kinase pathway, mitogen-activated protein kinase pathway. MEK1, mitogen-activated proteinkinase kinase 1. ERK1/2, extracellular signal-regulated kinases 1 and 2. PI3K, phosphoinositide 3-kinase. ATK, agammaglobulinemia tyrosine kinase. FoxO1a, forehead box O1a. ROS, reactive oxygen species. XIAP, X-linked inhibitor of apoptosis. NF-kB, nuclear factor kappa-light-chain-enhancer of activated B cells. E3 ligase, E3 ubiquitin ligase [25,27,32,35,36,37,38,39,43,44,45].

A growing body of preclinical studies suggests that copper is crucial for the advancement of metastatic cancer. Copper’s involvement may extend to tumour growth, proliferation, epithelial–mesenchymal transition, and the formation of both the tumour microenvironment and the pre-metastatic niche [34]. Copper was thought to exert a pivotal function in signalling pathways associated with receptor tyrosine kinase (RTK). The activated RTK then triggers the phosphorylation of downstream proteins like extracellular regulated protein kinases (ERK) and agammaglobulinemia tyrosine kinase (ATK), ultimately resulting in migration and proliferation of cancer cells [35]. Moreover, copper-induced ATK activation can subsequently accelerate the phosphorylation and redistribution within the cell of forehead box O1a (FoxO1a) and forehead box O4 (FoxO4). This process promotes tumour growth [32]. Furthermore, the amassment of this ion has been found to correlate with cancer angiogenesis [27]. It is showed that copper exhibits precise spatial regulation, moving from perinuclear regions of the cell towards the ends of extending filopodia and traversing the cell membrane into the extracellular space during angiogenic processes [36]. It has been shown that inflammatory cytokines, such as IL-17, drive copper uptake by cells through the induction of the metalloreductase six-transmembrane epithelial antigen of prostate-4 (STEAP4). IL-17-induced intracellular copper elevation leads to the activation of ubiquitin (E3) ligase and X-linked inhibitor of apoptosis protein (XIAP). Tumour metastasis is initiated by the copper uptake facilitated by STEAP4. Moreover colitis-associated colon tumorigenesis is also facilitated by the promotion of STEAP4 [37]. It needs to be added that the absorption of copper through high affinity copper uptake protein-1 (CTR1) triggers allosteric activation of mitogen-activated protein kinase (MEK1), enhancing oncogenic signalling through the mitogen-activated protein kinase (MAP kinase) pathway (Figure 2) [25]. Copper has the capacity to provoke oxidative stress (reactive oxygen species, ROS) by generating highly reactive hydroxyl radicals through the Fenton-like reactions [38]. Enhanced mitochondrial ROS production induced by copper leads to complete autophagy, thereby promoting cancer cell survival. This phenomenon has been confirmed by both in vitro and in vivo studies on cervical cancer [39]. Copper homeostasis is also controlled, among other factors, by the Golgi-localized ATPases transporting Cu, ATP7A, and ATP7B. Expanding tumours actively absorb copper and utilize ATP7A/B to control the presence of this metal for oncogenic enzymes like LOX and LOX-like proteins, enhancing the invasiveness of malignant cells. Additionally, the activity and movement of ATP7A/B enable tumour cells to detoxify certain drugs used in the chemotherapy of various solid tumours [40]. In recent research, a newly discovered type of cell death termed cuproptosis, dependent on copper, has been identified. This mechanism is distinct from all previously known pathways leading to cell death [41]. Cuproptosis takes place when copper binds to lipoylated enzymes in the tricarboxylic acid (TCA) cycle, resulting in the aggregation of proteins, proteotoxic stress, and, ultimately, cancer cell death [42]. Nevertheless, the influence of cuproptosis on malignant tumours remains incompletely comprehended from a clinical standpoint [30]. In investigations concerning the anticancer efficacy of copper (II) complexes and other metallic coordination compounds on breast and lung cancer cells, the copper (II) complex displayed encouraging cytotoxic potency. Its comparable toxicity to cisplatin, the standard chemotherapeutic agent, indicates its potential as an alternative anticancer therapeutic. Moreover, the copper (II) complex exhibited significant cytotoxicity against cancer cells, underscoring its potential utility in anticancer interventions [43]. Recent in vitro and in vivo studies have further elucidated that the anticancer activity of metal compounds, such as copper (II) complexes via intercalative mechanisms, is characterized by their ability to recognize, bind, and induce DNA damage in lung cancer cells treated with complex (2), thereby prompting apoptosis-mediated cell death [43,44]. Furthermore, the combination of the copper (II) complex with cisplatin, another chemotherapeutic agent, manifested synergistic effects, suggesting the promise of this combination as an efficacious anticancer therapeutic strategy [43,45].

### 3.3. Cobalt 

**Figure 3:**
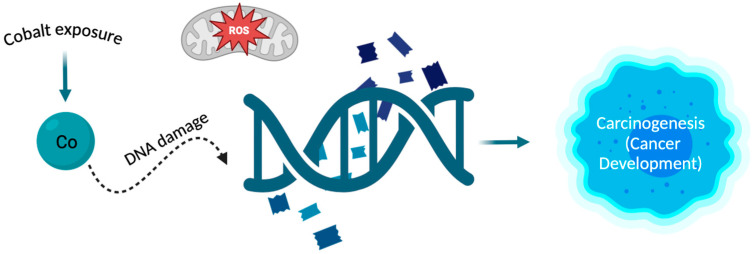
The relationship between cobalt and carcinogenesis. The cobalt atom can interact with DNA, and processes such as oxidative stress and DNA damage are the main mechanisms by which cobalt can induce carcinogenesis. Exposure to cobalt leads to changes in DNA which can result in the development of cancer [54,55].

Cobalt (Co) is a vital trace element for the human body, existing in both organic and inorganic forms [46]. The main purpose of cobalt in the human body revolves around its involvement in cobalamin (Cbl), also known as vitamin B12 [12]. That is why cobalt plays a vital role in the regulation of red blood cell production and why it is crucial to uphold adequate levels of cobalt in the human body, as a deficiency in this element can result in anaemia [47]. It also plays a significant role in the synthesis of amino acids and certain proteins in nerve cells and contributes to the production of neurotransmitters essential for the proper functioning of the body [46]. Cobalt is most recognized for its essential involvement in alkylcorrinoid cofactors, leveraging the distinctive characteristics of the cobalt-carbon bond to facilitate chemically intricate biotransformation’s [48]. It serves as the coenzyme for many crucial enzymes e.g., methylmalonic-CoA mutase or methionine synthase. These enzymes in humans play vital roles in maintaining health [12]. On the other hand, inorganic cobalt in ion form is toxic to the human body, and prolonged retention can lead to increasingly detrimental changes in cells [46]. Being exposed to it can result in conditions such as asthma, hard metal lung disease, contact allergy, and an elevated susceptibility to cancer [49]. Cobalt is commonly found in natural surroundings and can be generated because of human-related activities [46]. The primary route of cobalt absorption is through the respiratory system, although absorption through the skin is also possible [49]. People may encounter exposure to cobalt or its compounds in occupational settings where cobalt is used or manufactured, through cobalt-containing orthopaedic joint replacements, and from environmental sources [50]. Cobalt serves as the primary constituent in metal prostheses used in hip arthroplasty. Research indicates that metal particles, predominantly consisting of cobalt nanoparticles (CoNPs), can induce both systemic and local harmful reactions, attributed to various physical and chemical factors [51]. Biological toxicity was observed in cobalt nanoparticles, manifesting as the inhibition of osteoclast differentiation and proliferation across various concentrations [52]. Moreover, the study showed that nanoparticles of cobalt oxide have an impact on the electromechanical behaviour of heart muscle cells (cardiomyocytes) and the regulation of intracellular calcium. It needs to be added that these nanoparticles trigger the production of reactive oxygen species (ROS), resulting in oxidative stress. This oxidative stress may be linked to DNA damage and could negatively affect the functionality of cardiomyocytes [53]. Exposure of experimental animals to metallic cobalt or cobalt compounds resulted in tumours in rats and/or mice through various exposure routes and in several different tissue locations. Inhalation exposure to metallic cobalt or cobalt sulphate led to tumours in the lungs, pancreas, adrenal glands, and the hematopoietic system [50]. Alveolar/bronchiolar carcinomas in rodents, whether occurring spontaneously or due to chemical exposure, bear resemblance to a specific subtype of lung adenocarcinomas observed in humans. Oxidative stress is a key factor in pulmonary carcinogenesis induced by cobalt metal dust (CMD) in rodents, and these discoveries may have implications for understanding human lung cancers as well [54]. The impact of CoCl_2_ was also examined on numerous histone modifications at a global level. It was found that in both human lung cancer cells (A549) and human bronchial epithelial cells (Beas-2B), exposure to CoCl_2_ for 24 h increased the trimethylation of H3K4 and H3K27 through the activation of methyltransferases. Additionally, it elevated the trimethylation of H3K9 and H3K36 by inhibiting the histone demethylation process. It has been shown that cobalt ions disrupt the cellular epigenetic balance. Such changes could potentially result in modified gene expression patterns and contribute to carcinogenesis [55]. Moreover, it has been proven that the hypoxia-mimicking substance cobalt chloride (CoCl_2_) induces an elevation in the expression of chemokine (C-C motif) ligand-18 (CCL18) [56]. CCL18 serves as an indicator of the M2 macrophage subset, contributing to the immunosuppressive characteristics of the tumour microenvironment and playing a crucial role in cancer immune evasion. As a result, higher levels of CCL18 in both the bloodstream and the tumour are correlated with a poorer prognosis for the patient [57]. Furthermore, it has been confirmed that CCL18 also promotes the migration of endothelial cells and angiogenesis in breast cancer [58]. It has been noted that the available data from studies on humans are insufficient to assess the association between cancer in humans and exposure to cobalt and cobalt compounds. However, there is sufficient evidence of carcinogenicity from experimental animal studies and evidence from studies on the mechanisms of carcinogenesis, indicating that the release of cobalt ions is a crucial event influencing carcinogenicity (Figure 3) [50].

### 3.4. Iron 

Iron (Fe) stands as the most plentiful metal within the human body, and no autonomous life forms on Earth can thrive without it [59]. It is the predominant metal found in the human brain and a vital trace element that governs various cellular processes [60]. Most of the iron in the human body is found in red blood cells. Despite the high amount of iron in food, many individuals globally experience anaemia. Insufficient iron leads to a hindered synthesis of iron-containing proteins and hampers cell growth [61]. Iron is also necessary for the cellular proliferation as a cofactor of many enzymes [62]. While iron is essential for supporting cell growth and fundamental functions, it can also pose harm and carcinogenic risks [63]. Many medical disorders, like hemochromatosis, prolonged infections with hepatitis B and C, viruses and the presence of endometriosis, are identified as factors associated with an excess of iron, which increases the risk of developing cancer in humans [64]. Moreover, an environment with an excess of iron encourages cellular evolution to become resistant to ferroptosis, constituting a significant factor in the development of cancer [62]. Epidemiological research has shown a link between surplus iron levels and higher rates of cancer occurrence and susceptibility. Meanwhile, laboratory investigations have suggested that iron plays a role in the onset of cancer and fuelling tumour expansion [65]. Iron is essential in the advancement and spread of tumours, primarily because of its critical role in promoting the survival of tumour cells and restructuring the microenvironment within the tumour [66]. 

An abundance of iron is closely linked to the development of tumours in various human cancer types, operating through diverse mechanisms. These mechanisms encompass impacting signal transduction in cancer cells, influencing DNA replication, repair, and cell cycle advancement, catalysing the creation of mutagenic hydroxyl radicals, and serving as a vital nutrient for the growth of proliferating tumour cells [67]. It was discovered that accumulation of iron facilitated by mitochondria contributes to the development of cancer and the Warburg effect in osteosarcoma cells [68]. The Warburg Effect is characterized by an elevated rate of glucose consumption and the preferential generation of lactate, even when oxygen is present [69]. Simultaneously, inducing a deficiency of iron could emerge as an innovative and effective approach in treating osteosarcoma [68]. Findings also revealed that exposing colorectal cancer (CRC) cells to iron also stimulated the Warburg effect by triggering reactive oxygen species (ROS) and activating nuclear factor erythroid 2-related factor 2 (NRF2). Furthermore, this iron exposure demonstrated an increased resilience of CRC cells to ferroptosis [70]. It was also found that excessive iron accumulation in the liver resulting from F-box and leucine-rich repeat protein-5 (FBXL5) ablation increases the risk of hepatocellular carcinoma (HCC) progression. This condition triggers oxidative stress, tissue damage, inflammation, and compensatory proliferation of hepatocytes [71]. Other studies showed that an abundance of iron saturates the binding capacity of transferrin, leading to the formation of non-transferrin-bound iron (NTBI). This NTBI can initiate free-radical reactions, potentially contributing to oxidant-induced breast carcinogenesis. Additionally, the surplus iron and the disturbance of iron metabolism by local oestrogen in the breast contribute to the production of reactive oxygen species [72]. The data collected from the National Health and Nutrition Examination Survey I and the National Health Evaluation Follow-Up Study showed that increased iron consumption was linked to a heightened risk of colon cancer across the entire colon in both males and females [73], while in other sources we can find that the development of lung cancer is facilitated by iron-dependent cyclin-dependent kinase-1 (CDK-1) activity through the activation of the GP130/STAT3 signalling pathway [74]. 

Macrophages also play a pivotal role in maintaining iron balance; they capture it through the engulfment of aging red blood cells and serve as a significant reservoir of accessible iron in the body [75]. Considering the various ways in which macrophages have developed mechanisms to acquire, transport, store, and release iron, it can be hypothesized that tumour cells may influence or instruct these macrophages to provide iron, thereby facilitating the growth of the tumour [76]. Elevated concentrations of ferrous (Fe^2+^) iron have the potential to produce reactive oxygen species through Fenton chemistry reactions. These heightened levels can result in harm to mitochondria and genomic DNA, ultimately fostering the development of cancer [60]. It should be noted that accumulation of iron has been observed in tissues as they age and in diseases associated with the aging process [77]. This induction of senescence in the cancer microenvironment due to therapy is acknowledged as one of the factors that propel tumour advancement [78]. Additionally, an excess of iron might arise unintentionally in specific cancer patients, stemming from the treatment of symptomatic anaemia through inappropriate iron-restoration therapies. This occurs without prior assessment of the body’s iron status, and both conditions collaboratively contribute to the exacerbation of the tumour [79]. Moreover, various infections can also increase the risk of cancer development. Reactive oxygen/nitrogen species produced during inflammation not only harm DNA but also impact other large biological molecules like proteins and lipids, leading to impaired functionality. Transferrin, when oxidatively damaged, releases iron ions that can potentially initiate Fenton reactions, producing more reactive oxygen species in the process [80].

To support their growth, cancer cells demonstrate a heightened requirement for iron when compared to normal, non-cancerous cells. This reliance on iron renders cancer cells more susceptible to iron-induced cell death, known as ferroptosis [81]. It is a recently identified type of controlled necrotic cell death and has been shown to be significant in various conditions [82]. Ferroptosis is implicated in cancer development and could potentially serve as a valuable approach for anti-cancer treatment. Various pieces of evidence indicate that ferroptosis is pivotal in inhibiting tumorigenesis [59]. 

Moreover, the discussion on the role of Fe should include the function of the transferrin receptor and its overexpression in cancer cells, which meets the heightened Fe demand in these cells. Additionally, it should address the efforts to target this receptor for anticancer therapy. Malignant cells frequently overexpress TfR1 due to its pivotal role in cancer cell pathology, and this heightened expression is often linked to poor prognosis across various cancer types. The increased TfR1 levels on malignant cells, combined with its extracellular availability, capacity for internalization, and crucial role in cancer pathology, render this receptor a promising target for antibody-mediated therapy [83].

Furthermore, new research has provided insights into the involvement of iron metabolism in cancer stem cells (CSC). These findings propose that selectively addressing iron metabolism in CSCs could enhance the effectiveness of cancer therapy [84]. The important role in anti-cancer therapy is also played by iron chelators, which have exhibited strong anti-cancer properties in various types of cancers, as evidenced in both laboratory cell culture studies and clinical trials [85].

### 3.5. Manganese 

Manganese (Mn) is a vital metal found abundantly in the environment and is crucial for various essential processes within the human body. Its importance lies in being incorporated into protein structures, acting as a necessary cofactor. Without manganese, crucial functions such as immune response, energy regulation, growth, blood clotting, and the body’s ability to manage oxidative stress by-products would be greatly compromised [86,87]. Manganese in its divalent form (Mn(II)), acting as a cofactor for mitochondrial superoxide dismutase (MnSOD), helps eliminate oxygen free radicals like superoxide and hydroxyl radicals. This role is vital for maintaining a balance between oxidative and antioxidative processes, shielding against oxidative stress and its harmful effects. Additionally, studies suggest that at lower concentrations, Mn(II) may protect against the toxic effects of cadmium (Cd) by virtue of its antioxidative properties and its ability to hinder the uptake of this heavy metal into cells [88].

Within cells, free radicals form naturally as part of cellular functions like mitochondrial processes, alongside being generated by external factors such as exposure to ionizing radiation. An imbalance between the creation of free radicals and the body’s capacity to counteract them with antioxidants leads to oxidative stress, causing harm to vital cellular structures—a phenomenon termed oxidative damage. This interplay of free radicals, oxidative stress, and oxidative damage is widely acknowledged in various diseases, notably cancer. Beyond their traditional role in cancer development via DNA mutation and genomic instability, free radicals activate pathways that support cell growth, survival, and blood vessel formation, all of which contribute to tumour progression [89]. Manganese superoxide dismutase (MnSOD) significantly influences cancer development owing to its capability to scavenge reactive oxygen species (ROS) [90]—manganese superoxide dismutase serves as the initial defence mechanism against reactive oxygen species ROS by facilitating the conversion of two superoxide molecules into oxygen and hydrogen peroxide (H2O2) [91,92]. This process involves a cyclic exchange of reduction and oxidation reactions at the active metal site [91].

MnSOD has the capacity to directly influence several signalling pathways leading to cell death in cancer, such as apoptosis, proptosis, and autophagy. Elevated levels of MnSOD expression are linked to increased resistance to chemotherapy and radiation therapy across various cancer types. Recently, the focus has shifted towards exploring posttranslational modifications of MnSOD, particularly its acetylation at lysine residue 68, shedding light on its crucial roles in advancing cancer progression. As the significance of the immune system in cancer development garners increased attention, the role of MnSOD in the tumour’s immune microenvironment has emerged as a crucial focus. Growing evidence suggests that immune cells infiltrating tumours play a pivotal role in cancer progression, and their activity is closely linked to MnSOD expression across various cancer types. A positive correlation was discovered between MnSOD expression, CXCL8 levels, and neutrophil infiltration, indicating the involvement of the “MnSOD-CXCL8-neutrophil recruitment” pathway in cancer advancement. Additionally, studies have revealed a positive association between MnSOD expression and the infiltration of CD68+ macrophages, potentially indicating unfavourable outcomes in inflammation-induced lung adenocarcinoma. Moreover, heightened MnSOD expression has been noted in aggressive triple-negative breast cancer (TNBC) cases, suggesting it as an adverse prognostic indicator [93]. Assessing the levels of SOD and peroxide-eliminating enzymes is vital for comprehending cellular variations in response to vectors promoting SOD overexpression. Additionally, longstanding evidence indicates that malignant cell lines typically exhibit lower MnSOD levels compared to their normal or non-malignant counterparts [94]. Furthermore, there is ongoing exploration into nanoparticles with potential applications in cancer detection. For instance, in a study conducted by Du et al. (2020), a bi-modal nanopericle-targeted probe for prostate cancer was developed. This probe demonstrated specific accumulation within prostate cancer cells and tumour tissues using optical imaging and MRI in a preclinical model. In vitro and in vivo imaging outcomes suggest that Mn-Msn-Cy7 nanoprobes, which target prostate-specific membrane antigen (PSA), hold promise for detecting prostate cancer [95]. 

**Figure 4:**
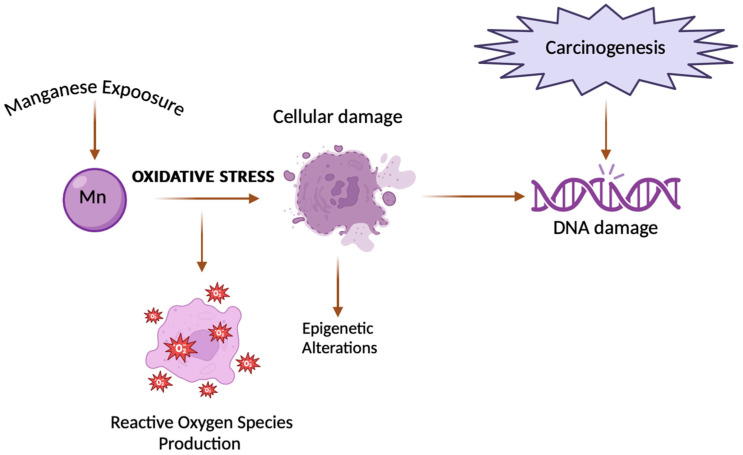
Link between manganese and carcinogenesis. The figure shows how exposure to manganese can lead to DNA damage, which in turn can trigger carcinogenic processes, leading to the development of cancer [92,94].

As previously mentioned, at lower concentrations, Mn(II) may protect against the toxic effects of cadmium (Cd) by virtue of its antioxidative properties and its ability to hinder the uptake of this heavy metal into cells [88]. Prior exposure to small amounts of Mn(II) has been shown to increase resistance to cadmium (Cd)−related fatalities and liver damage in both mice and rats. Studies conducted on rat liver mitochondria exposed to cadmium suggest that Mn(II) ions might shield against Cd-triggered lipid peroxidation and the suppression of antioxidant enzymes [96]. Cadmium (Cd) pollution has emerged as a significant worldwide issue, given its extensive presence and severe toxicity, posing a serious risk to both human and animal well-being [97]. According to the International Agency for Research on Cancer (IARC), cadmium falls under the category of Group I carcinogens. Numerous epidemiological studies have consistently identified Cd as a significant risk factor for the development of lung cancer [98]. Nevertheless, the relationship between cadmium exposure and the emergence of tumours in alternate locations like the kidney, breast, and prostate may also hold considerable importance. Moreover, the heightened likelihood of cancer may extend beyond occupations characterized by elevated exposure levels and could also manifest due to environmental factors, such as proximity to locales involved in the processing of heavy metals [99]. Information demonstrates the crucial role of Mn(II) and its protective effect against the Cd−inducted cytotoxic impacts and carcinogenesis (Figure 4).

### 3.6. Other Heavy Metals

Many other metals share certain common mechanisms of tumour formation, yet each also possesses distinct pathways of its own. Many metal carcinogens such as arsenic, cadmium, beryllium or mercury have been observed to generate reactive oxygen species (ROS) and elevate oxidative stress levels [5]. Additionally, cadmium and arsenic similarly induce many processes that may stimulate carcinogenesis. They disrupt the action of antioxidants such as glutathione (GSH), aggravating the cellular antioxidant capacity [5,100]. It should be noted that these elements competitively interact with or substitute crucial metals like zinc and calcium within proteins, serving as a primary mechanism of cytotoxicity within the cell [5,101]. Moreover, they inhibit cell autophagy, which is critical for tumour suppression. Arsenic reduces SLBP levels and subsequently leads to aberrant polyadenylation of canonical histone mRNA, thus promoting carcinogenesis. Research revealed that Nickel (Ni) also triggered a reduction in SLBP through a similar mechanism [5,100,101,102]. Ni is also primarily observed to stimulate hypoxia-induced signalling pathways by competing with iron in prolyl hydroxylase [5,103]. Studies have shown that arsenic can induce genotoxicity by disrupting DNA repair and causing chromosomal instability in the cell, which may lead to mutation of tumour suppressor genes such as p53 [104]. It also causes double strand breaks, leading to chromosomal aberrations. On the other hand, the analysis indicated that beryllium metal is improbable to function as a conventional non-threshold mutagen. While effects on DNA repair and cellular transformation were noted, their significance in vivo requires additional investigation. The correlation between beryllium exposure and its potential to cause cancer is still being debated within the scientific community, with ongoing research efforts underway [5,105]. Exposure to mercury is linked to cancer risk, although conflicting data exist. Both cancerous and healthy tissues accumulate mercury differently, potentially contributing to tumour development. Mercury’s influence on cell proliferation and its impact on various signalling pathways suggest mechanisms for promoting carcinogenesis. Additionally, the oxidative DNA damage, genotoxicity, and epigenetic effects of mercury may play roles in cancer development [106,107]. There is a possibility that metals may also potentially instigate tumorigenesis through a confluence of their impacts. A thorough grasp of the mechanisms underlying the onset of metal-induced cancer can offer valuable perspectives for therapeutic strategies targeting molecular pathways involved in metal-induced carcinogenesis.

## 4. Conclusions 

Heavy metals play a significant role in cancer formation. Numerous evidence suggests that exposure to heavy metals, may contribute to cancer initiation and progression through various mechanisms. Heavy-metal-induced carcinogenesis itself involves a complex interaction of genetic, epigenetic, and molecular mechanisms. These pathways often intersect and interact, leading to increased carcinogenicity. 

It is essential to underscore the critical role of chemical speciation in the biological activity of metals. For instance, Cr (VI) is recognized as a human carcinogen, whereas Cr (III), once thought to be an essential element, likely has no distinct biological function [108]. Metal salts, such as chlorides, used in cell and animal experiments are quickly transformed in biological media into mixtures of complexes with biological ligands, including proteins, which significantly alters their activity [109]. Furthermore, metal speciation in the environment dictates their bioavailability and toxicity to living organisms, including humans [110]. It is also crucial to note that the mechanisms of pro- or anti-cancer activities of soluble metal complexes are likely very different from those of metal nanoparticles [111]. Research on the connections between metal nanoparticles and cancer is rapidly advancing, though this topic is largely beyond the scope of this review.

The carcinogenic potential of heavy metals is dose-dependent, with higher levels of exposure correlating with increased cancer risk. Long-term exposure, even at low concentrations, can cumulatively increase the risk of cancer. Given the widespread presence of heavy metals in the environment and industrial processes, limiting exposure is critical to cancer prevention. This includes implementing stringent regulations, adopting safer industrial practices, and promoting public awareness of potential sources of exposure. Like early detection, due to the latency period between heavy metal exposure and cancer manifestation, early detection strategies are essential to ensure timely intervention and treatment. Surveillance programs targeting high-risk populations can facilitate early diagnosis and improve outcomes. 

It is also important to note that metals play a crucial role not only in cancer development but also in the formation of metastases. Metals significantly influence the metastatic process by affecting the function of various proteins and enzymes. Notably, an excess of essential metals such as iron and copper are frequently linked to both carcinogenesis and metastatic disease [112].

It is important to distinguish that essential trace elements (Fe, Cu, Zn, Mn, Co, Mo) naturally contribute to cancer prevention or can promote cancer development when their metabolism is dysregulated, which is the primary focus of this article. Conversely, metal compounds can also cause cancer through environmental or industrial exposure. However, this latter topic has been extensively covered in the literature, unlike the specific area we have chosen to examine.

Although significant progress has been made in understanding the carcinogenicity of heavy metals, knowledge gaps remain, particularly regarding the molecular mechanisms underlying their carcinogenic effects. Further research is needed to elucidate these mechanisms and develop targeted interventions for cancer prevention and treatment. 

In summary, solving the problem related to the impact of heavy metals on carcinogenesis processes requires a multi-faceted approach including:

(1) Continuous monitoring of environment with increased levels of harmful substances for humans;
(2) Standard use of protective equipment in accordance with procedures outlined by legal regulations and public health initiatives;
(3) Ongoing monitoring of the health of individuals exposed to the harmful effects of various factors (harmful elements) present in the environment, conducting periodic (standard and additional) examinations;
(4) Early implementation of medical procedures to prevent disease development, limiting the possibility of metastasis;
(5) Establishing a procedural algorithm depending on the diagnosed disease and the impact of the harmful compound on the human body.

The above review of the literature on carcinogenesis provides a detailed overview of the current knowledge on the role of heavy metals in carcinogenesis. Additionally, it highlights areas where research is lacking or where results are inconsistent. Suggests potential directions for future research to address these gaps. Informs you about risk assessments and regulatory guidance on heavy metals.

It is a valuable source of information for researchers, professionals, and students wishing to understand the relationship between heavy metals and cancer.

## 5. Study Limitations 

Although a comprehensive review of carcinogenesis provides valuable insights, it also has several limitations. The vast amount of research available may make it impossible to comprehensively cover all relevant research, so the authors focused on specific aspects. Carcinogenesis involves many interacting factors, which makes it difficult to isolate the specific impact of heavy metals. Genetic and environmental differences among individuals may influence susceptibility to heavy metal-induced carcinogenesis, complicating generalizations.

## Data Availability

Not applicable.
